# Prognostic and clinicopathologic significance of SIRT1 expression in hepatocellular carcinoma

**DOI:** 10.18632/oncotarget.14096

**Published:** 2016-12-22

**Authors:** Hongyuan Jiang, Xiao Zhang, Yuquan Tao, Liang Shan, Qijun Jiang, Yongchun Yu, Feng Cai, Lifang Ma

**Affiliations:** ^1^ Shanghai Municipal Hospital of Traditional Chinese Medicine Affiliated to Shanghai TCM University, Shanghai, 200071, China; ^2^ Department of Clinical Laboratory Medicine, Shanghai Tenth People's Hospital of Tongji University, Shanghai, 200072, China; ^3^ Department of Clinical Laboratory Medicine, Shanghai Municipal Hospital of Traditional Chinese Medicine Affiliated to Shanghai TCM University, Shanghai, 200071, China

**Keywords:** meta-analysis, prognosis, clinical value, HCC, hazard ratio

## Abstract

The clinical value of SIRT1 in hepatocellular carcinoma (HCC) remains controversial. This meta-analysis was performed to investigate the prognostic and clinicopathological significance of the histone deacetylase SIRT1 in HCC. Pooled hazard ratios (HRs) for survival outcomes and pooled odds ratios (ORs) for clinical parameters associated with SIRT1 were calculated in nine studies using Review Manager. Meta-analysis showed that increased SIRT1 expression is associated with poor overall survival (OS) (HR=1.82, 95% confidence interval (CI): 1.49-2.22, P<0.00001) and disease-free survival (DFS) (HR=1.44, 95%CI: 1.06-1.96, P=0.02) in HCC. Increased expression of SIRT1 is more common in female than male HCC patients (OR=0.47, 95%CI: 0.32-0.70, P=0.0001). The increased SIRT1 expression correlates with hepatitis B virus (HBV) infection (OR=1.63, 95%CI: 1.04-2.57, P=0.03), large tumor size (OR=1.81, 95%CI: 1.05-3.13, P=0.03), high p53 expression (OR=2.71, 95%CI: 1.39-5.29, P=0.003), high levels of alpha-fetoprotein (AFP; cutoff value: 400 ng/ml, OR=1.84, 95%CI: 1.26-2.69, P=0.002), and tumor stage (OR=1.72, 95%CI: 1.27-2.32, P=0.0004). Re-sampling statistics for 5,000,000 samples revealed that increased SIRT1 expression is associated with higher TNM stage (OR=1.70, 95%CI: 1.69-1.70, P<0.00001). These results indicate that SIRT1 is a new biomarker off HCC as well as a potentially effective therapeutic target.

## INTRODUCTION

Primary liver cancer is the sixth most commonly occurring cancer in the world, and the second largest contributor to cancer mortality [1]. Most (70% to 90%) primary liver cancers are hepatocellular carcinoma (HCC) [2], the third leading cause of cancer-related death [3]. Most HCC risk factors (chronic infection with hepatitis B (HBV) and/or C virus (HCV) and alcoholic liver disease) operate by promoting the development of cirrhosis [4]. The treatment options for HCC are limited, mainly because of the lack of reliable biomarkers.

Silent information regulator 1 (SIRT1) is a member of mammalian sirtuin protein family, which are histone deacetylases that utilize NAD+ as a cofactor [5, 6]. SIRT1 promotes or inhibits many biological processes, including regulation of gene expression, cellular metabolism, stress response, aging, and chemo-resistance [7]. Importantly, SIRT1 is thought to promote HCC tumorigenesis. Recent studies have demonstrated that compared with normal liver or surrounding tumor tissues, SIRT1 is strongly overexpressed in human HCC [6, 7]. SIRT1 facilitates HCC metastasis by promoting peroxisome proliferator-activated receptor coactivator 1α (PGC-1α)-mediated mitochondrial biogenesis [8]. In experimental HCC mouse models or HCC cell lines, SIRT1 overexpression promotes metastasis through epithelial-mesenchymal transition (EMT) [9]. In HCC, SIRT1 can activate telomerase reverse transcriptase (TERT) gene promoter [10], promote YAP/TEAD4 association [11], and stabilize c-Myc protein [12]. Moreover, microRNA-133b can inhibit HCC progression by directly targeting SIRT1 [13]. SIRT1 inhibition enhances the antitumor effect of doxorubicin [7], cisplatin [14], and irradiation [15]. Recent studies have indicated that increased SIRT1 expression is associated with poor HCC prognosis [7–9, 16–21]. Therefore, we speculated that there might be a significant correlation between the expression of SIRT1 and the clinical outcomes of HCC.

In order to more accurately evaluate the association between the SIRT1 expression and HCC clinical outcomes, we performed this meta-analysis to explore the value of SIRT1 as a potential clinical HCC biomarker.

## RESULTS

### Characteristics of included studies

**Figure 1:**
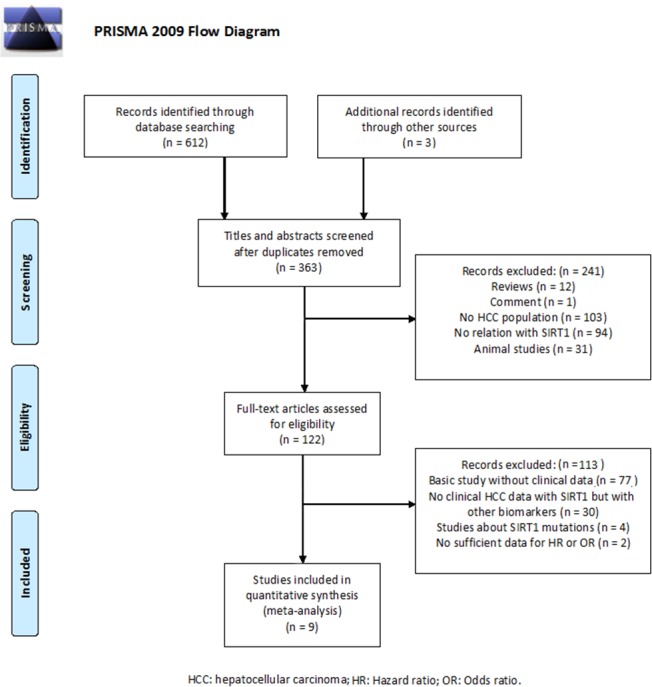
Flow diagram of literature search and selection

During primary literature search, 145 studies in PubMed, 228 studies in EMBASE, 115 studies in Web of Science, 68 studies in OVID, and 56 studies in Cochrane Library were found. In Oncomine and The Cancer Genome Atlas (TCGA) database, no data about SIRT1 amplification changes in HCC were reported (Supplementary Figure S1). 363 studies remained after excluding duplicate studies. 354 studies were excluded because they contained no relevant survival or clinical data (Figure 1). Finally, nine studies were included in our meta-analysis (Table 1) [7–9, 16–21]. The studies were published from 2011 to 2016, and included 1435 patients from Asia. High expression of SIRT1 was present in 54.2% of the patients. Eight studies used IHC to examine the SIRT1 expression, and one study used western blotting. Eight studies reported overall survival (OS) data and three studies reported disease free survival (DFS) data. Eight studies reported the association between SIRT1 high expression and poor prognosis in HCC. Five of these eight studies reported a significant association (P<0.05). The detailed information is displayed in Supplementary Table S1.

**Table 1:** Characteristics of included studies in the meta-analysis

Study	Year	Total subject (Male/Female)	Age	High expression	Low expression	TNM stage	Method	Follow-up time (month)	Type of Survival data
Chen [7]	2012	172(142/30)	55.9(mean)	95	77	I-III	IHC	125	OS
Li [8]	2016	72(65/7)	50.1(mean)	41	31	I-III	IHC	60	DFS OS
Hao [9]	2014	99(89/10)	≤50 55 patients >50 44 patients	76	23	I-IV	IHC	130	OS
Song [16]	2014	300(267/33)	53.0(mean)	155	145	I-IV	IHC	64	OS
Choi [17]	2011	90(77/13)	<60 48 patients ≥60 42 patients	50	40	I-IV	IHC	NR	NR
Jang [18]	2012	154(132/22)	<55 66 patients ≥55 88 patients	55	99	I-IV	IHC	140	DFS OS
Zhang [19]	2015	252(NR)	NR	153	98	I-IV	Western Blotting	125	DFS OS
Cheng [20]	2015	148(NR)	NR	77	71	I-III	IHC	80	OS
Liu [21]	2016	148 (128/20)	<50 93 patients ≥50 55 patients	76	72	I-III	IHC	80	OS

Abbreviations: NR, no report; IHC, immunohistochemistry; OS, overall survival; DFS, disease-free survival.

### Meta-analysis for prognostic value

**Figure 2:**
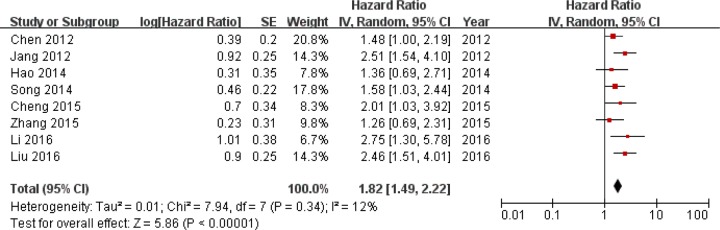
Forest plot of HRs for the association of SIRT1 expression in HCC with OS

**Figure 3:**
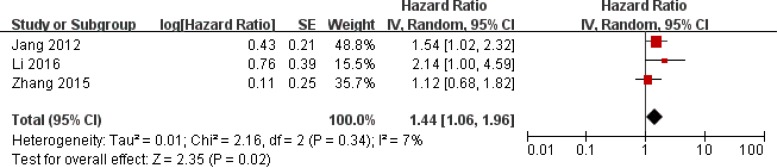
Forest plot of HRs for the association of SIRT1 expression in HCC with DFS

The combined analysis of 8 studies showed that high expression of SIRT1 was associated with poor prognosis in OS in HCC patients (hazard Ratio (HR)=1.82, 95% confidence interval (CI): 1.49-2.22, P<0.00001) without significant heterogeneity (I^2^ =12%, P=0.34) (Figure 2). As for 3 studies reporting DFS, increased SIRT1 expression was also associated with poor HCC prognosis (HR=1.44, 95%CI:1.06-1.96, P=0.02) without significant heterogeneity (I^2^ =7%, P=0.34) (Figure 3).

### Quality assessment

The quality of the studies was assessed by the Newcastle-Ottawa Quality Assessment Scale (NOS) (http://www.ohri.ca/programs/clinical_epidemiology/nosgen.pdf) [22]. Three studies with scores higher than 6 were deemed as high quality studies (Supplementary Table S2).

### Subgroup analysis

The results of subgroup analysis are summarized in Table 2. The subgroup analysis was performed according to publication year, subjects in study, the length of follow-up time, and quality of the studies. Except for the association between SIRT1 expression and the patients in the subgroup, the difference between subgroups could be also calculated by Review Manager. High SIRT1 expression was associated with poor HCC prognosis both in studies before 2015 (HR=1.69, 95%CI: 1.31-2.18, P<0.0001), and in studies published between 2015 and 2016 (HR=2.03, 95%CI: 1.45-2.84, P<0.0001). No significant difference between subgroups was found (P=0.39). As for the subjects in the study, no significant difference between subgroups was found (Subjects <150: HR=2.13, 95%CI: 1.56-2.90, P<0.00001; Subjects ≥150: HR=1.66, 95%CI: 1.27-2.17, P=0.0002; Subgroup difference: P=0.24). Regarding the association between follow-up time and prognosis, high SIRT1 expression was associated with poor HCC prognosis both in studies with follow-up time <100 months (HR=2.03, 95%CI: 1.55-2.66, P<0.00001) and in studies with follow-up time≥100 months (HR=1.63, 95%CI: 1.20-2.22, P<0.002); no significant difference was observed (P=0.39). As for the three high quality studies, the high SIRT1 expression was associated with poor HCC prognosis (HR=1.76, 95%CI: 1.29-2.39, P=0.0003). Moreover, as for the other five studies, high SIRT1 expression was also associated with poor HCC prognosis (HR=1.90, 95%CI: 1.40-2.57, P<0.0001); no significant difference between subgroups was found (P=0.73).

**Table 2:** A summary of hazard ratios (HRs) for the subgroup analyses of SIRT1 expression in HCC patients

Subgroups	Patients number	HR	95%CI	P value	Subgroup differences
Studies before 2015	725	1.69	1.31-2.18	<0.0001	P=0.39
Studies in 2015 and 2016	620	2.03	1.45-2.84	<0.0001	
Subjects <150	467	2.13	1.56-2.90	<0.00001	P=0.24
Subjects ≥150	878	1.66	1.27-2.17	0.0002	
Follow-up <100 months	668	2.03	1.55-2.66	<0.00001	P=0.30
Follow-up ≥100 months	677	1.63	1.20-2.22	0.002	
high quality studies	626	1.76	1.29-2.39	0.0003	P=0.73
other studies	719	1.9	1.40-2.57	<0.0001	

Abbreviations: HR, hazard ratio; CI, confidence interval.

### Sensitivity analysis

Sensitivity analysis was performed by omitting one study at a time and calculating the pooled HRs again [19]. In each sensitivity group, the P value for testing the statistical significance of the association was not changed and heterogeneity was not observed (I^2^ <50%, P>0.1). Thus, the stability of the meta-analysis was not influenced by any single independent study (Table 3).

**Table 3:** The influence of individual study on the pooled estimate (OR) for overall survival

Studies omitted	Year	HR	95%CI	P value	Heterogeneity	
					I2(%)	P value
None		1.82	1.49-2.22	<0.00001	12	0.34
Chen [7]	2012	1.92	1.54-2.40	<0.00001	9	0.36
Li [8]	2016	1.76	1.44-2.16	<0.00001	10	0.35
Hao [9]	2014	1.87	1.51-2.31	<0.00001	17	0.3
Song [16]	2014	1.88	1.48-2.37	<0.00001	20	0.28
Jang [18]	2012	1.71	1.40-2.09	<0.00001	0	0.43
Zhang [19]	2015	1.89	1.54-2.31	<0.00001	7	0.38
Cheng [20]	2015	1.8	1.44-2.26	<0.00001	23	0.27
Liu [21]	2016	1.72	1.40-2.11	<0.00001	3	0.4

Abbreviations: HR, hazard ratio; CI, confidence interval.

### Publication bias

Publication bias of the included studies was evaluated by Begg's funnel plot and Egger's test. The detailed results are displayed in Table 4. Since all P values were greater than 0.05, no significant publication bias was present.

**Table 4:** Begg’s funnel plot and Egger’s test of publication bias on the relationships between miR-200c and prognostic value in cancer

	Patient number	Begg's funnel plot	P value	Egger's test	
		Z test for plot asymmetry		t value	P value
OS					
Overall	1435	0.25	0.803	0.48	0.645
Studies before 2015	725	0.34	0.734	0.06	0.961
Studies in 2015 and 2016	620	−0.34	1	−0.09	0.934
Subjects <150	467	0.34	0.734	−0.5	0.665
Subjects ≥150	878	−0.34	1	0	0.999
Follow-up <100 months	668	0.34	0.734	1.12	0.381
Follow-up ≥100 months	677	−0.34	1	−0.32	0.782
DFS					
Overall	478	0	1	0.59	0.659

Abbreviations: OS, overall survival; DFS, disease-free survival.

### Clinicopathologic analysis

Eight studies were included in the clinicopathologic analysis. Higher expression of SIRT1 was more common in female HCC patients than male patients (odds ratio (OR)=0.47, 95%CI: 0.32-0.70, P=0.0001) (Supplementary Figure S2). In addition, increased SIRT1 expression was a more common phenomenon in HBV-infected HCC patients than in non-HBV HCC patients (OR=1.63, 95%CI: 1.04-2.57, P=0.03) (Supplementary Figure S3). Higher expression of SIRT1 was associated with larger tumor size (OR=1.81, 95%CI: 1.05-3.13, P=0.03) (Supplementary Figure S4), and with increased expression of p53 (OR=2.71, 95%CI: 1.39-5.29, P=0.003) (Supplementary Figure S5). Moreover, higher expression of SIRT1 was associated with increased alpha-fetoprotein (AFP) levels. When the cutoff value was set to 400 ng/ml, the association was significant (OR=1.84, 95%CI: 1.26-2.69, P=0.002) (Supplementary Figure S6). However, when the cutoff value was set to 100 ng/ml, no association was found (OR=1.67, 95%CI: 0.91-3.05, P=0.09) (Supplementary Figure S7). Increased SIRT1 expression was also associated with the increased TNM stage (stage III-IV vs stage I-II, OR=1.91, 95%CI: 1.12-3.26, P=0.02) (Supplementary Figure S8). However, when evaluating cirrhosis and age, no significant association was found (Supplementary Figures S9 and S10).

### Re-sampling statistics

**Figure 4:**
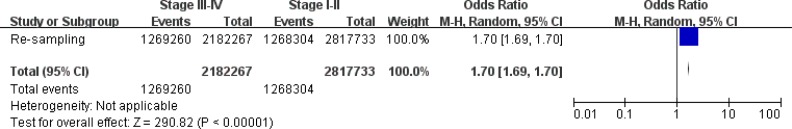
Meta-analysis evaluating SIRT1 expression for TNM stage in 1000 re-sampling groups containing five million samples

Bootstrap re-sampling procedures were applied to investigate the association between SIRT1 expression and TNM stage in order to get robust and reproducible results [23] (Supplementary Excel File S1). One randomly generated results are summarized in Supplementary Excel File S2. The distribution of odds ratios was between 1.43 and 2.045, suggesting that the increased SIRT1 expression is associated with higher TNM stage. When evaluating 5,000,000 samples, the odds ratio was 1.70 (95%CI: 1.69-1.70, P<0.00001) (Figure 4).

## DISCUSSION

SIRT1, homologue of the yeast Sir2 protein, is the most researched sirtuin in the mammalian sirtuin family [24]. SIRT1 activates transcription silencing, DNA repair, recombination of ribosomal DNA, nuclear receptors to stimulate mitochondrial biogenesis, circadian clock, and lipid homeostasis [25]. In HCC, SIRT1 is aberrantly overexpressed and activates TERT gene promoter [10], YAP/TEAD4 association [11], c-Myc stabilization [12], NF-kB [26], wnt/β-catenin [13], and PI3K/AKT [27] signaling to stimulate cell proliferation and metastasis [7, 28, 29]. Moreover, SIRT1 induces expression of the transcription factor SOX2 to stimulate the self-renewal of liver cancer stem cells [21]. Due to the importance of SIRT1 in biological processes and HCC progression, it is urgent to uncover the mechanisms of how SIRT1 regulates the HCC progression, and to evaluate the clinical value of SIRT1. In our meta-analysis, we analyzed eight studies in Asian population between 2012 and 2016, and found that increased SIRT1 expression correlated with poor prognosis in HCC patients (HR=1.82, 95%CI: 1.49-2.22, P<0.00001).

Deacetylase activity of SIRT1 is essential for the SIRT1 oncogenic function in HCC. When the deacetylation domain of SIRT1 is mutated, HCC cell proliferation and colony formation are inhibited [7]. For instance, LC3 can be deacetylated by SIRT1 to induce autophagy in HepG2 cells [30]. Moreover, p53 is the most widely studied target of SIRT1, and p53 deacetylation by SIRT1 can repress cellular senescence and apoptosis, thus stimulating tumorigenesis in HCC [5]. Inactivated SIRT1 (no phosphorylation on Ser 47) can bind to mutated p53, thus activating AMPK/mTOR pathway to exert the carcinogenic effects in HCC [19]. Our clinicopathologic analysis indicates that increased expression of SIRT1 correlates with high expression of p53 in HCC (OR=2.71, 95%CI: 1.39-5.29, P=0.003). Hepatitis B virus (HBV) is one of the main causes of HCC [31]. SIRT1 is upregulated in HBV-infected cells, and its transcription and replication can be upregulated by SIRT1 in HCC [32, 33]. In our clinicopathologic analysis, we also found that the increased SIRT1 expression was more common in HBV-infected HCC (OR=1.63, 95%CI: 1.04-2.57, P=0.03).

Although we have comprehensively analyzed the prognostic and clinicopathologic value of SIRT1 in HCC, some limitations remain in our study. First, the SIRT1 expression was analyzed by IHC or western blotting, with no unified standard or cutoff value. In the study by Chen et. al [7], dark brown nuclear staining in more than 5% of cancer cells was defined as positive. In the study by Hao et. al [9] and Choi et. al [17], dark brown nuclear staining in more than 10% of cancer cells was defined as positive. In the study by Jang et. al [18], dark brown nuclear staining in more than 30% of cancer cells was defined as positive. In the study by Song et. al [16], H-score system was used, and more than 30% of positively stained cells was defined as positive. In the study by Li et. al [8], the definition of SIRT1 positive expression was not clearly stated. In other studies [19–21], the standards were not reported. Second, the sample size in the subgroup analysis was not big enough; thus, the statistic power was limited. Third, no study with Caucasian population was included in our meta-analysis. Although there are multiple studies about SIRT1 in HCC performed by European or American scientists, these studies focus on molecular mechanisms, and not enough clinical samples were tested.

Nevertheless, this is the first meta-analysis study that indicates that increased SIRT1 expression is associated with poor prognosis in HCC. Moreover, we found that SIRT1 expression is associated with sex, HBV-infection, AFP levels, tumor size, TNM stage, and p53 expression. Our study indicates that SIRT1 expression might serve as a potential therapeutic target and prognostic marker in HCC.

## MATERIALS AND METHODS

### Literature search

We followed the PRISMA statement in our meta-analysis (Supplementary Checklist S1). PubMed, EMBASE, Web of Science, Cochrane Library and OVID databases were searched since their inception up to October 9th 2016, without language and publication restrictions. The key words of the search were (“Sirtuin 1 [MeSH]” OR “SIR2” OR “SIR2L1” OR “SIR2alpha” OR “silent mating type information regulation 2 homolog 1” OR “NAD-dependent deacetylase sirtuin-1” OR “sirtuin (silent mating type information regulation 2 homolog) 1” OR “SIRT1 protein, human”OR “SIR2L1 protein, human” OR “sirtuin 1, human” OR “Sir2-like 1 protein, human” OR “sirtuin (silent mating type information regulation 2 homolog) 1 (S. cerevisiae), human” OR “SIRT1” OR “SIRT 1” OR “SIRT-1” OR “Sirtuin 1” OR “Sirtuin-1” OR “Silent information regulator 1”) AND (“Liver Neoplasms [MeSH]” OR “Carcinoma, Hepatocellular [MeSH]” OR “liver cancer” OR “hepatocarcinoma” OR “hepatocellular carcinoma” OR “hepatocellular cancer” OR “HCC” OR “liver AND cancer” OR “liver AND neoplasms”). We also screened review articles [5, 34, 35] and their reference lists to complete our research. Oncomine (User ID: 1zhangxiao@tongji.edu.cn) and TCGA (analyzed by cBioPortal [36, 37]) were searched to make our data complete. H. Jiang and X. Zhang independently searched the databases, excluded the irrelevant studies with double check and disagreements were resolved by consensus of all the authors. All retrieved articles were managed by the EndNote X6 software (available at the website www.endnote.com, Thomson Reuters).

### Selection criteria

The articles were included if they: (1) Proved prognostic or clinicopathologic value of the SIRT1 expression in HCC; (2) More than 30 patients were enrolled in studies; (3) Studies provided sufficient data to obtain the odds ratio (OR) or hazard ratio (HR) and 95% confidence intervals (CI). H. Jiang and X. Zhang scanned the titles and abstracts to exclude irrelevant studies, and the full articles were examined by all authors in detail. No overlapping patient populations were included in our meta-analysis.

### Data extraction

H. Jiang and X. Zhang independently extracted the following data: first author, year of publication, race of publication, number of patients with SIRT1 high expression and low expression, method of SIRT1 expression, follow-up time and type of survival data. Since multivariate analysis takes confounding factors into consideration and is more accurate, it would be selected when univariate and multivariate analysis were both present in the study [38]. If HR was not reported in the study, Engauge Digitizer version 4.1 (free software down-loaded from http://sourceforge.net) was used to read the Kaplan-Meier survival curves to obtain the HRs and their 95% CIs by two different authors (H. Jiang and X. Zhang). If the essential data was not reported in the study, we asked corresponding authors for additional information.

### Quality assessment

The Newcastle-Ottawa Quality Assessment Scale (NOS) (http://www.ohri.ca/programs/clinical_epidemiology/nosgen.pdf) was used to assess the quality of the study [22]. The score was from 0 to 9, and the study with a score higher than 6 was deemed as a high quality study.

### Statistical analysis

Hazard ratios (HRs) and corresponding 95% confidence intervals (CIs) were combined to evaluate the value of SIRT1 expression on HCC prognosis. An HR>1 suggested poor prognosis in patients with high expression of SIRT1. P value <0.05 suggested significant association. The association between SIRT1 expression and clinicopathology significance for HCC was measured by odds ratios (ORs) and 95% CIs. We extracted the data from Kaplan-Meier survival curve using Engauge-Digitizer version 7.2 if there was no direct data in the study [39]. Two authors (H. Jiang and X. Zhang) checked the curves independently to reduce reading variability. Cochran's Q test and Higgins I-squared statistic were used to measure the heterogeneity among the studies. I^2^ ≥50% and P value for Cochran's Q test <0.1 suggested significant heterogeneity. Random-effects models were selected to avoid the influence of heterogeneity. These statistical analyses were performed by Review Manager Version 5.1 software(http://ims.cochrane.org/revman) and the publication bias was measured by R (http://cran.r-project.org/bin/windows/base). Only the biomarkers that more than two studies reported could be included in our clinicopathologic analysis. Bootstrap re-sampling procedure was used to validate the association between SIRT1 expression and TNM stage as we described previously [23]. The re-sampling statistic program was displayed in Supplementary Excel File S1. A randomly produced result, the ORs containing all samples and the ORs distribution of each re-sample group were shown in Supplementary Excel File S2.

## SUPPLEMENTARY MATERIALS FIGURES AND TABLES










