# Supplementary material for: Prognostic and clinicopathologic significance of SIRT1 expression in hepatocellular carcinoma
Source: Oncotarget. 2016 Dec 22;8(32):52357–65. doi: 10.18632/oncotarget.14096 (PMC5581034; doi:10.18632/oncotarget.14096)
Supplement: Supplementary file 1 [file oncotarget-08-52357-s001.pdf]

Prognostic and clinicopathologic significance of SIRT1 expression in hepatocellular carcinoma

SUPPLEMENTARY FIGURES AND TABLES

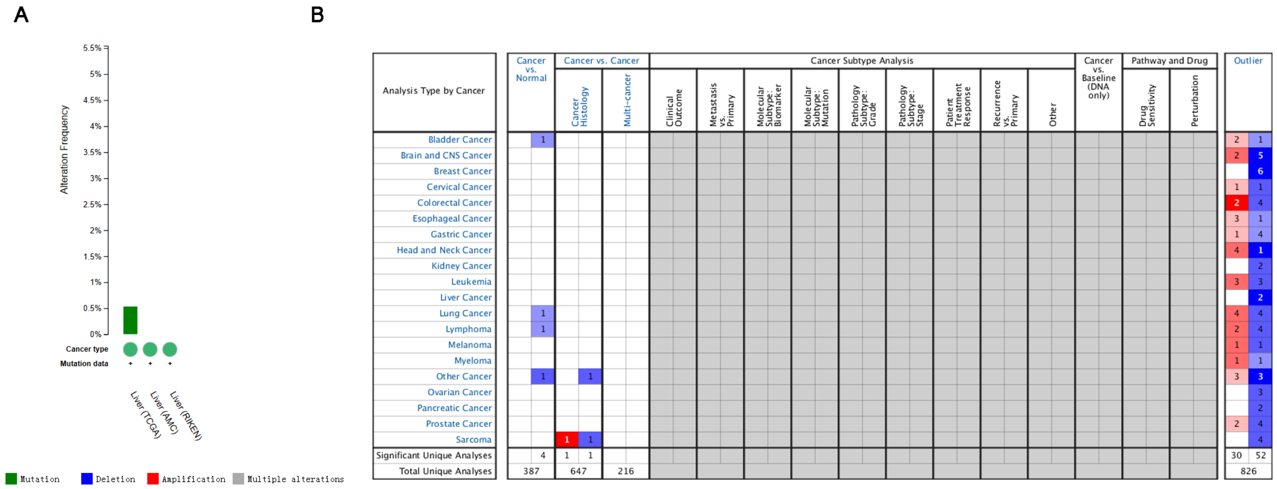

Supplementary Figure S1: The results for Oncomine and TCGA database search. A. The results for Oncomine database search. **B.** The results for TCGA database search.

Study or Subgroup	Male Events	Male Total	Female Events	Female Total	Weight	Odds Ratio M-H, Random, 95% CI
Chen 2012	71	142	24	30	16.3%	0.25 [0.10, 0.65]
Cheng 2015	38	72	10	15	10.8%	0.56 [0.17, 1.80]
Choi 2011	39	77	11	13	6.0%	0.19 [0.04, 0.90]
Hao 2014	67	89	9	10	3.3%	0.34 [0.04, 2.82]
Jang 2012	47	132	8	22	16.8%	0.97 [0.38, 2.47]
Li 2016	37	65	4	7	6.0%	0.99 [0.21, 4.79]
Liu 2016	62	128	14	20	14.3%	0.40 [0.15, 1.11]
Song 2014	124	267	21	33	26.4%	0.50 [0.23, 1.05]
**Total (95% CI)**		**972**		**150**	**100.0%**	**0.47 [0.32, 0.70]**
Total events	485		101			

Heterogeneity: $Tau^2 = 0.00$; $Chi^2 = 6.46$, df = 7 (P = 0.49); $I^2 = 0\%$

Test for overall effect: Z = 3.81 (P = 0.0001)

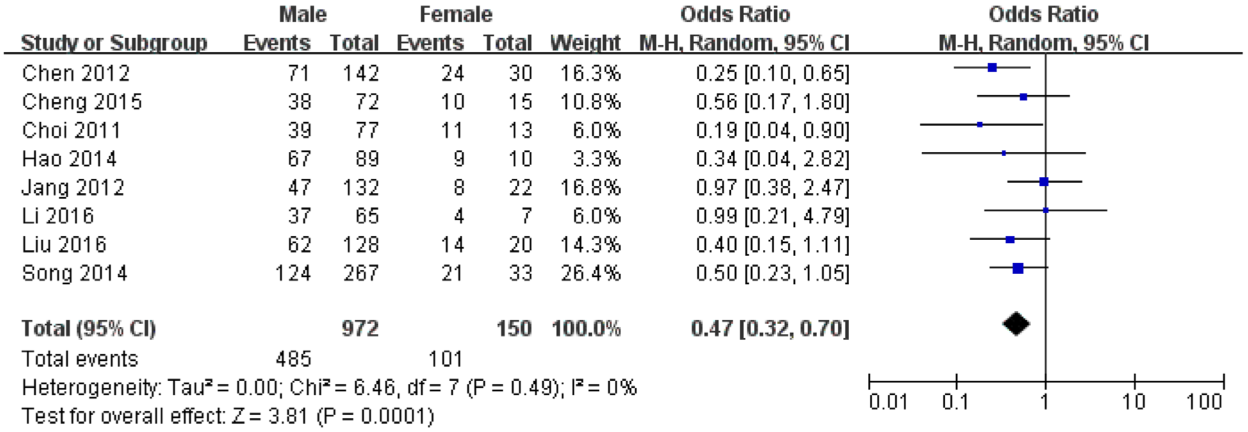

Supplementary Figure S2: Meta-analysis evaluating SIRT1 expression for sex in HCC.

	HBV		non-HBV			Odds Ratio
Study or Subgroup	Events	Total	Events	Total	Weight	M-H, Random, 95% CI
Jang 2012	46	112	9	42	30.1%	2.56 [1.12, 5.85]
Li 2016	36	61	5	11	12.3%	1.73 [0.47, 6.29]
Song 2014	122	247	23	53	57.6%	1.27 [0.70, 2.31]
**Total (95% CI)**		**420**		**106**	**100.0%**	**1.63 [1.04, 2.57]**
Total events	204		37			

Heterogeneity: Tau² = 0.00; Chi² = 1.80, df = 2 (P = 0.41); I² = 0%
Test for overall effect: Z = 2.11 (P = 0.03)

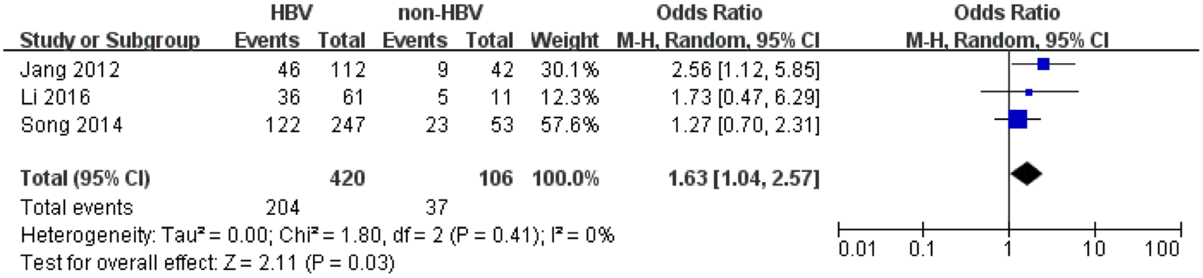

Supplementary Figure S3: Meta-analysis evaluating SIRT1 expression for HBV infection in HCC.

	>5cm		≤5cm			Odds Ratio
Study or Subgroup	Events	Total	Events	Total	Weight	M-H, Random, 95% CI
Chen 2012	55	102	40	70	26.5%	0.88 [0.48, 1.62]
Cheng 2015	36	59	7	23	16.5%	3.58 [1.28, 10.03]
Hao 2014	38	44	38	55	16.4%	2.83 [1.01, 7.97]
Li 2016	29	44	12	28	17.6%	2.58 [0.97, 6.83]
Liu 2016	52	101	17	40	23.0%	1.44 [0.69, 3.00]
**Total (95% CI)**		**350**		**216**	**100.0%**	**1.81 [1.05, 3.13]**
Total events	210		114			

Heterogeneity: Tau² = 0.19; Chi² = 8.21, df = 4 (P = 0.08); I² = 51%

Test for overall effect: Z = 2.14 (P = 0.03)

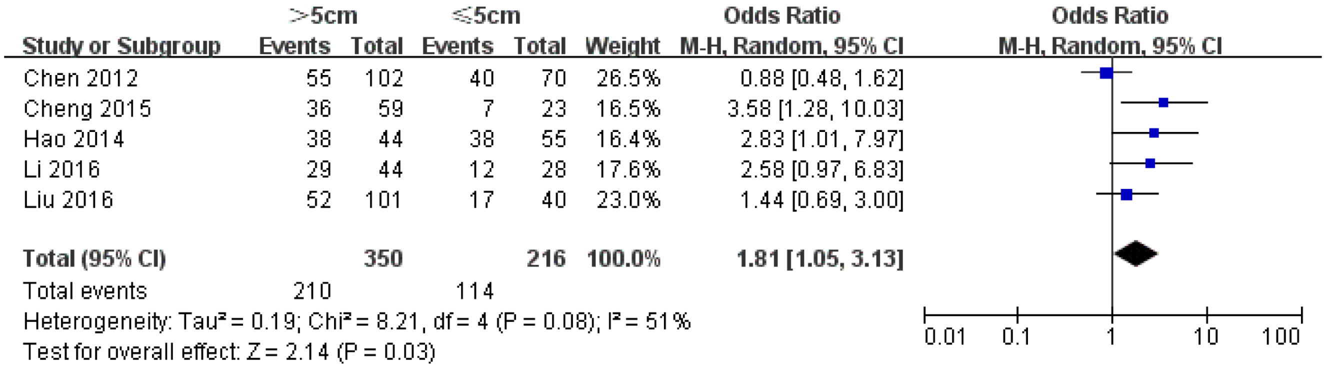

Supplementary Figure S4: Meta-analysis evaluating SIRT1 expression for tumor size in HCC.

	P53 positive		P53 negative			Odds Ratio
Study or Subgroup	Events	Total	Events	Total	Weight	M-H, Random, 95% CI
Choi 2011	20	27	30	63	25.6%	3.14 [1.16, 8.48]
Jang 2012	18	27	37	127	28.9%	4.86 [2.00, 11.81]
Song 2014	62	109	83	191	45.4%	1.72 [1.07, 2.76]
**Total (95% CI)**		**163**		**381**	**100.0%**	**2.71 [1.39, 5.29]**
Total events	100		150			

Heterogeneity: $Tau^2 = 0.20$; $Chi^2 = 4.58$, df = 2 (P = 0.10); $I^2 = 56\%$

Test for overall effect: Z = 2.92 (P = 0.003)

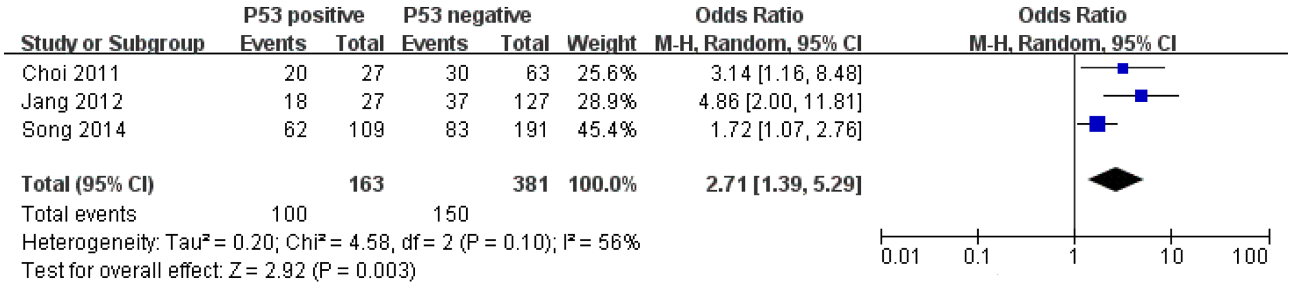

Supplementary Figure S5: Meta-analysis evaluating SIRT1 expression for p53 expression in HCC.

	AFP ≥400		AFP <400			Odds Ratio
Study or Subgroup	Events	Total	Events	Total	Weight	M-H, Random, 95% CI
Choi 2011	16	21	34	69	11.7%	3.29 [1.09, 9.99]
Hao 2014	33	40	41	57	14.4%	1.84 [0.68, 5.00]
Li 2016	17	27	24	45	15.1%	1.49 [0.56, 3.95]
Song 2014	55	96	83	190	58.7%	1.73 [1.05, 2.84]
**Total (95% CI)**		**184**		**361**	**100.0%**	**1.84 [1.26, 2.69]**
Total events	121		182			

Heterogeneity: Tau² = 0.00; Chi² = 1.30, df = 3 (P = 0.73); I² = 0%

Test for overall effect: Z = 3.14 (P = 0.002)

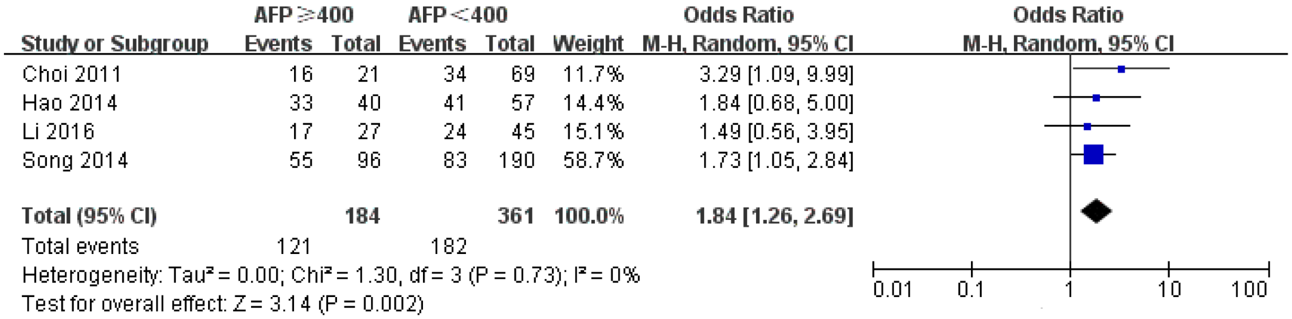

Supplementary Figure S6: Meta-analysis evaluating SIRT1 expression for AFP level (cutoff value: 400 ng/ml) in HCC.

	AFP ≥100		AFP <100			Odds Ratio
Study or Subgroup	Events	Total	Events	Total	Weight	M-H, Random, 95% CI
Jang 2012	25	53	30	101	62.9%	2.11 [1.06, 4.20]
Li 2016	21	36	20	36	37.1%	1.12 [0.44, 2.85]
**Total (95% CI)**		**89**		**137**	**100.0%**	**1.67 [0.91, 3.05]**
Total events	46		50			

Heterogeneity: Tau² = 0.03; Chi² = 1.15, df = 1 (P = 0.28); I² = 13%

Test for overall effect: Z = 1.67 (P = 0.09)

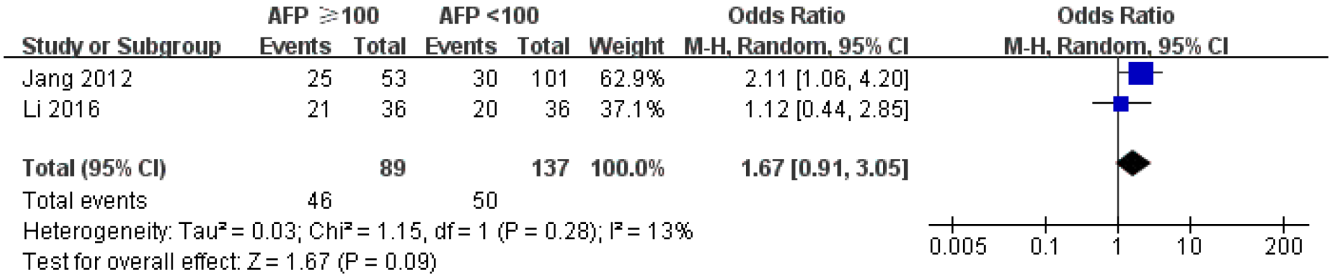

Supplementary Figure S7: Meta-analysis evaluating SIRT1 expression for AFP level (cutoff value: 100 ng/ml) in HCC.

	stage III and IV		stage I and II			Odds Ratio
Study or Subgroup	Events	Total	Events	Total	Weight	M-H, Random, 95% CI
Cheng 2015	31	43	17	44	17.4%	4.10 [1.67, 10.10]
Hao 2014	30	34	46	65	13.0%	3.10 [0.96, 10.00]
Jang 2012	12	36	43	118	19.7%	0.87 [0.40, 1.92]
Liu 2016	45	71	31	77	22.5%	2.57 [1.32, 4.99]
Song 2014	82	160	63	140	27.4%	1.28 [0.82, 2.03]
**Total (95% CI)**		**344**		**444**	**100.0%**	**1.91 [1.12, 3.26]**
Total events	200		200			

Heterogeneity: Tau² = 0.22; Chi² = 10.38, df = 4 (P = 0.03); I² = 61%
Test for overall effect: Z = 2.36 (P = 0.02)

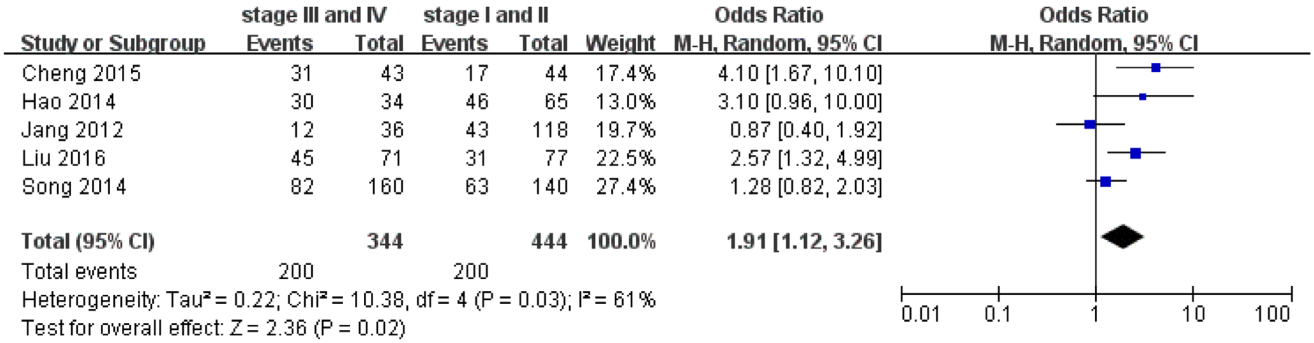

Supplementary Figure S8: Meta-analysis evaluating SIRT1 expression for TNM stage in HCC.

Study or Subgroup	Cirrhosis present Events	Cirrhosis present Total	Cirrhosis absent Events	Cirrhosis absent Total	Weight	Odds Ratio M-H, Random, 95% CI
Hao 2014	26	35	50	64	27.0%	0.81 [0.31, 2.12]
Jang 2012	31	81	24	73	56.6%	1.27 [0.65, 2.46]
Li 2016	36	59	5	13	16.4%	2.50 [0.73, 8.60]
**Total (95% CI)**		**175**		**150**	**100.0%**	**1.25 [0.76, 2.07]**
Total events	93		79			

Heterogeneity: Tau² = 0.00; Chi² = 2.01, df = 2 (P = 0.37); I² = 0%
Test for overall effect: Z = 0.89 (P = 0.37)

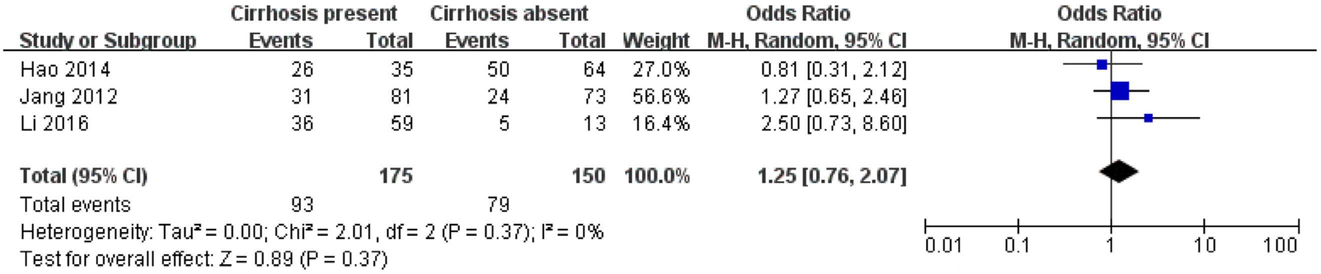

Supplementary Figure S9: Meta-analysis evaluating SIRT1 expression for cirrhosis in HCC.

	≥50		<50			Odds Ratio
Study or Subgroup	Events	Total	Events	Total	Weight	M-H, Random, 95% CI
Cheng 2015	14	24	30	59	29.0%	1.35 [0.52, 3.53]
Hao 2014	36	44	40	55	28.6%	1.69 [0.64, 4.45]
Liu 2016	24	55	52	93	42.3%	0.61 [0.31, 1.20]
**Total (95% CI)**		**123**		**207**	**100.0%**	**1.03 [0.53, 1.98]**
Total events	74		122			

Heterogeneity: Tau² = 0.15; Chi² = 3.54, df = 2 (P = 0.17); I² = 43%

Test for overall effect: Z = 0.09 (P = 0.93)

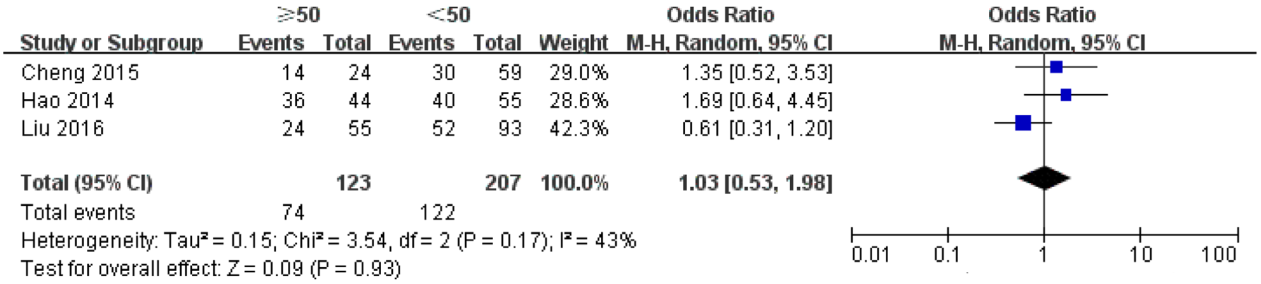

Supplementary Figure S10: Meta-analysis evaluating SIRT1 expression for age in HCC.

Supplementary Table S1: The association between SIRT1 expression and HCC prognosis in each study

Study	Year	HR	95% CI	P value
Chen [7]	2012	1.48	1.00-2.19	0.05
Li [8]	2016	2.75	1.30-5.78	0.008
Hao [9]	2014	1.36	0.69-2.71	0.38
Song [16]	2014	1.58	1.03-2.44	0.04
Jang [18]	2012	2.51	1.54-4.10	0.0002
Zhang [19]	2015	1.26	0.69-2.31	0.46
Cheng [20]	2015	2.01	1.03-3.92	0.04
Liu [21]	2016	2.46	1.51-4.01	0.0003

Abbreviations: HR, hazard ratio; CI, confidence interval.

Supplementary Table S2: Study quality assessment based on the NEWCASTLE - OTTAWA QUALITY ASSESSMENT SCALE

See Supplementary File 1

Supplementary Checklist S1: PRISMA Checklist. Preferred Reporting Items for Systematic Reviews and Meta-Analyses: The PRISMA Statement

See Supplementary File 2

Supplementary Excel File S1: Re-sampling program evaluating SIRT1expression and TNM stage in HCC

See Supplementary File 3

Supplementary Excel File S2: Re-sampling results of SIRT1 expression and TNM stage in HCC

See Supplementary File 4
