# Supplementary material for: Prognostic and clinicopathologic significance of SIRT1 expression in hepatocellular carcinoma
Source: Oncotarget. 2016 Dec 22;8(32):52357–65. doi: 10.18632/oncotarget.14096 (PMC5581034; doi:10.18632/oncotarget.14096)
Supplement: Supplementary file 2 [file oncotarget-08-52357-s002.docx]

**Supplementary Table 2.** **Study quality assessment based on the NEWCASTLE - OTTAWA QUALITY ASSESSMENT SCALE**

| NEWCASTLE - OTTAWA QUALITY ASSESSMENT SCALE COHORT STUDIES | | | | | | | | |
| --- | --- | --- | --- | --- | --- | --- | --- | --- |
|  | Song [16] 2014 | Li [8] 2016 | Chen [7] 2012 | Jang [18] 2012 | Zhang [19] 2015 | Hao [9] 2014 | Cheng [20] 2015 | Liu [21] 2016 |
| **Selection** |  |  |  |  |  |  |  |  |
| 1) Representativeness of the exposed cohort |  |  |  |  |  |  |  |  |
| a) truly representative of the average level in the community * | √ |  | √ | √ |  |  |  | √ |
| b) somewhat representative of the average level in the community * |  |  |  |  |  | √ | √ |  |
| c) selected group of users eg nurses, volunteers |  |  |  |  |  |  |  |  |
| d) no description of the derivation of the cohort |  | √ |  |  | √ |  |  |  |
|  |  |  |  |  |  |  |  |  |
| 2) Selection of the non-exposed cohort |  |  |  |  |  |  |  |  |
| a) drawn from the same community as the exposed cohort * | √ | √ | √ | √ | √ | √ | √ | √ |
| b) drawn from a different source |  |  |  |  |  |  |  |  |
| c) no description of the derivation of the non-exposed cohort |  |  |  |  |  |  |  |  |
|  |  |  |  |  |  |  |  |  |
| 3) Ascertainment of exposure |  |  |  |  |  |  |  |  |
| a) secure record (eg surgical records) * | √ | √ | √ | √ | √ | √ | √ | √ |
| b) structured interview * |  |  |  |  |  |  |  |  |
| c) written self-report |  |  |  |  |  |  |  |  |
| d) no description |  |  |  |  |  |  |  |  |
|  |  |  |  |  |  |  |  |  |
| 4) Demonstration that outcome of interest was not present at start of study |  |  |  |  |  |  |  |  |
| a) yes * | √ | √ | √ | √ | √ | √ |  | √ |
| b) no |  |  |  |  |  |  | √ |  |
|  |  |  |  |  |  |  |  |  |
| Comparability |  |  |  |  |  |  |  |  |
| 1) Comparability of cohorts on the basis of the design or analysis |  |  |  |  |  |  |  |  |
| a) study controls for the most important factor * | √ | √ | √ | √ | √ | √ | √ | √ |
| b) study controls for any additional factor * (This criteria could be modified to indicate specific control for a second important factor.) * |  |  |  | √ | √ |  |  |  |
|  |  |  |  |  |  |  |  |  |
| Outcome |  |  |  |  |  |  |  |  |
| 1) Assessment of outcome |  |  |  |  |  |  |  |  |
| a) independent blind assessment * |  |  | √ |  |  |  |  |  |
| b) record linkage * |  |  |  |  |  |  |  |  |
| c) self-report |  |  |  |  |  |  |  |  |
| d) no description | √ | √ |  | √ | √ | √ | √ | √ |
|  |  |  |  |  |  |  |  |  |
| 2) Was follow-up long enough for outcomes to occur |  |  |  |  |  |  |  |  |
| a) yes (select an adequate follow up period for outcome of interest) * | √ | √ | √ | √ | √ | √ | √ | √ |
| b) no |  |  |  |  |  |  |  |  |
|  |  |  |  |  |  |  |  |  |
| 3) Adequacy of follow up of cohorts |  |  |  |  |  |  |  |  |
| a) complete follow up - all subjects accounted for * |  |  |  |  |  |  |  |  |
| b) subjects lost to follow up unlikely to introduce bias * | √ |  | √ |  |  |  |  |  |
| c) low follow up rate and no description of those lost |  |  |  |  |  |  |  |  |
| d) no statement |  | √ |  | √ | √ | √ | √ | √ |
|  |  |  |  |  |  |  |  |  |
| Scores | 7 | 5 | 8 | 7 | 6 | 6 | 5 | 6 |

Note: A study can be awarded a maximum of one asterisk for each numbered item within the Selection and Outcome categories. A maximum of two asterisks can be given for Comparability. Studies more than 6 scores were deemed as high quality studies.
